# Supplementary material for: A model for understanding the causes and consequences of walking impairments
Source: PLoS One. 2022 Dec 28;17(12):e0270731. doi: 10.1371/journal.pone.0270731 (PMC9797092; doi:10.1371/journal.pone.0270731)
Supplement: S1 Appendix — (DOCX) [file pone.0270731.s002.docx]

Appendix 1

### Path 0: Condition → Diagnosis

Because of a loss of nerve cells in the substantia nigra, the patient was diagnosed as having Parkinson’s disease.

Note that this path is included for completeness but does not affect any downstream calculations due to the use of Diagnosis as a surrogate.

Path 1: Diagnosis → History

Because of osteoarthritis at the hip, the patient underwent a total hip arthroplasty.

Note: Diagnosis 🡪 ______ paths exist to (mostly) account for unmeasured Structure and Function variables. For example, if we measured cartilage thickness, we would add the “Cartilage Erosion” to Structure and Function, and the specific example above would be accounted for through Diagnosis 🡪 Structure and Function 🡪 History.

Path 2: Diagnosis → Structure and Function

Because of cerebral palsy secondary to periventricular leukomalacia, the patient developed spasticity in their plantarflexors.

Path 3: Diagnosis → Gait Mechanics

Because of an acquired brain injury, the patient’s vision was poor, resulting in slow walking.

Path 4: Diagnosis → Energy

Because of their Cushing’s Disease, the patient’s net energy consumption was low.

Path 5: Diagnosis → Mobility

Because of the patient’s peripheral neuropathy, walking was painful, leading to a sedentary lifestyle.

Path 6: Diagnosis → Quality of Life

Because of the patient’s genetic disorder, their expected lifespan was shortened, leading to depression.

Path 7: History → Structure and Function

Because the patient previously underwent a femoral derotation osteotomy, their femoral anteversion was 20$^{\circ}$.

Path 8: Structure and Function → Gait Mechanics

Because the patient had a forefoot adduction deformity, they walked with an internal foot progression angle.

Path 9: Structure and Function → Energy

Because the patient was obese their net energy consumption demands for walking were elevated.

Note: Dimensionless net energy consumption does not use lean mass as a normalizing constant, thus body fat percentage deviations from normal can skew the result. Keep in mind that we account for Gait Mechanics mediated effects on energy through Structure and Function 🡪 Gait Mechanics 🡪 Energy (e.g., obesity 🡪 wide base of support 🡪 high energy)

Path 10: Structure and Function → Mobility

Because the patient was weak, they had difficulty getting up from a seated position.

Note that we account for Gait Mechanics mediated effects on mobility through Body Structure 🡪 Gait Mechanics 🡪 Mobility (e.g., weakness 🡪 crouch 🡪 difficulty getting on a bus).

Path 11: Structure and Function → Quality of Life

Because the patient was weak, they had a low self-image.

Note that we account for gait mechanics mediated quality-of-life effects through Gait Mechanics 🡪 Quality of Life (e.g., brain injury 🡪 stiff knee gait 🡪 self-consciousness about appearance).

Path 12: Gait Mechanics → Energy

Because patient walked in a deep crouch their net energy consumption was elevated.

Note that the detailed mechanisms between “crouch” and “elevated net energy consumption” are not explicitly modeled. This is a model granularity choice.

Path 13: Gait Mechanics → Mobility

Because patient had limited speed, they had trouble keeping up with their peers.

Note that Gait Mechanics strictly means only the mechanics of free-speed, level-ground walking that we measure in our gait analysis testing. Walking is not the same as stair climbing (for example), even though they both use similar activation patterns and exhibit similar motions. Patients have common causes of impairments in gait and stair climbing (e.g., weakness). However, it is important to distinguish impairment due to common causes from impairment due to a direct cause.

Gait Mechanics 🡪 Mobility $\neq$ Gait Mechanics 🡨 Body Structure 🡪 Mobility.

As an explicit example, we wouldn’t expect walking impairments to cause bicycle riding impairments – but both are both caused (in part) by poor motor control and are thus correlated.

Path 14: Gait Mechanics → Quality-of-Life

Because the patient walked with inward pointing toes, they were unhappy about their appearance.

Path 15: Energy → Mobility

Because the patient had elevated walking energy, they were unable to walk long distances.

Path 16: Energy → Quality-of-Life

Because the patient had elevated walking energy, they were unable to join their friends in shopping, causing them to feel unhappy.

Path 17: Mobility → Quality-of-Life

Because the patient had limited mobility, they were reliant on others to perform many tasks for them (loss of independence).

Path 18 – Path 23: Age → __________

- **History** – because the patient was young, they did not undergo foot surgery.
- **Structure and Function** – because the patient was young, their femoral anteversion was high.
- **Gait Mechanics** – because the patient was young, they used short steps and fast cadence, even after accounting for stature [1]
- **Energy** – because the patient was young their resting energy was elevated [2].
- **Mobility** – because the patient was young (thus inexperienced), they had trouble riding on an escalator.

Note that trouble riding an escalator due to inexperience is different from trouble riding an escalator due to weakness or poor balance, which would be accounted for in the Structure and Function à Mobility path.

- **Quality-of-Life** – because the patient was going through puberty, they were anxious.

Path 24: Sex → History

Because the patient was a boy, he was less likely to get treatment and follow-up due to biases in how our society treats the sexes.

Path 25: Sex → Diagnosis

Because the patient was a boy, he was more likely to have cerebral palsy.

Path 26: Sex → Structure and Function

Because the patient was a girl, she had more femoral anteversion.

References

1. Vaughan CL, Langerak NG, O’Malley MJ. Neuromaturation of human locomotion revealed by non-dimensional scaling. Exp Brain Res. 2003;153: 123–127. doi:10.1007/s00221-003-1635-x

2. Maffeis C, Schutz Y, Micciolo R, Zoccante L, Pinelli L. Resting metabolic rate in six- to ten-year-old obese and nonobese children. The Journal of Pediatrics. 1993;122: 556–562. doi:10.1016/S0022-3476(05)83535-8
