# Supplementary material for: A model for understanding the causes and consequences of walking impairments
Source: PLoS One. 2022 Dec 28;17(12):e0270731. doi: 10.1371/journal.pone.0270731 (PMC9797092; doi:10.1371/journal.pone.0270731)
Supplement: S2 Appendix — (DOCX) [file pone.0270731.s003.docx]

| Appendix 2  Variables included in BART Models | | | | |  |
| --- | --- | --- | --- | --- | --- |
| Example | Exposure Node | Outcome Variable | Effect Type | Variables (Adjustment + Exposure) | |
| 1 | Structure and Function | Mean stance foot progression | Total | Lower Extremity Strength, Lower Extremity Static Motor Control, Lower Extremity Spasticity, Dynamic Motor Control, Diagnosis, Age, Max Hip Flex, Max Hip Exten, Max Hip Abd (Knee Extended), Popliteal Angle (Unilateral), Max Knee Flex, Max Knee Exten, Extensor Lag, Max Ankle Dorsiflex (Knee Extended), Max Ankle Plantarflex, Bimalleolar Axis Angle, Forefoot Var/Val, Forefoot Abd/Add, Hindfoot Var/Val, Midfoot Cavus/Planus, Troch Prominence Test Angle | |
| 2 | Structure and Function | FAQt | Total | Lower Extremity Strength, Lower Extremity Static Motor Control, Lower Extremity Spasticity, Dynamic Motor Control, Diagnosis, Age, Max Hip Flex, Max Hip Exten, Max Hip Abd (Knee Extended), Popliteal Angle (Unilateral), Max Knee Flex, Max Knee Exten, Extensor Lag, Max Ankle Dorsiflex (Knee Extended), Max Ankle Plantarflex, Bimalleolar Axis Angle, Forefoot Var/Val, Forefoot Abd/Add, Hindfoot Var/Val, Midfoot Cavus/Planus, Troch Prominence Test Angle | |
| 3 | Gait Mechanics | Net energy consumption | Total | Lower Extremity Strength, Lower Extremity Static Motor Control, Lower Extremity Spasticity, Dynamic Motor Control, Diagnosis, Mean Pelvic Tilt, Range-of-Motion Pelvic Tilt, Min. Stance Hip Flexion, Mean Stance Hip Rotation, Init. Contact Knee Flexion, Min. Stance Knee Flexion, Range-of-Motion Knee Flexion, Max. Swing Knee Flexion, Mean Stance Knee Rotation, Init. Contact Ankle Dorsiflexion, Mean Stance Ankle Dorsiflexion, Mean Swing Ankle Dorsiflexion, Mean Stance Foot Progression, Normalized Step Length, Normalized Cadence, Age, Max Hip Flex, Max Hip Exten, Max Hip Abd (Knee Extended), Popliteal Angle (Unilateral), Max Knee Flex, Max Knee Exten, Extensor Lag, Max Ankle Dorsiflex (Knee Extended), Max Ankle Plantarflex, Troch Prominence Test Angle, Bimalleolar Axis Angle, Forefoot Var/Val, Forefoot Abd/Add, Hindfoot Var/Val, Midfoot Cavus/Planus | |
| 4 | Age | Net energy consumption | Direct | Lower Extremity Strength, Lower Extremity Static Motor Control, Lower Extremity Spasticity, Dynamic Motor Control, Diagnosis, Mean Pelvic Tilt, Range-of-Motion Pelvic Tilt, Min. Stance Hip Flexion, Mean Stance Hip Rotation, Min. Stance Knee Flexion, Init. Contact Knee Flexion, Range-of-Motion Knee Flexion, Max. Swing Knee Flexion, Mean Stance Knee Rotation, Init. Contact Ankle Dorsiflexion, Mean Stance Ankle Dorsiflexion, Mean Swing Ankle Dorsiflexion, Mean Stance Foot Progression, Normalized Step Length, Normalized Cadence, Max Hip Flex, Max Hip Exten, Max Hip Abd (Knee Extended), Popliteal Angle (Unilateral), Max Knee Flex, Max Knee Exten, Extensor Lag, Max Ankle Dorsiflex (Knee Extended), Max Ankle Plantarflex, Troch Prominence Test Angle, Bimalleolar Axis Angle, Forefoot Var/Val, Forefoot Abd/Add, Hindfoot Var/Val, Midfoot Cavus/Planus, Age | |
| 5 | Structure and Function | Activities of Daily Living and Independence | Total | Lower Extremity Strength, Lower Extremity Static Motor Control, Lower Extremity Spasticity, Dynamic Motor Control, Diagnosis, Age, Max Hip Flex, Max Hip Exten, Max Hip Abd (Knee Extended), Popliteal Angle (Unilateral), Max Knee Flex, Max Knee Exten, Extensor Lag, Max Ankle Dorsiflex (Knee Extended), Max Ankle Plantarflex, Bimalleolar Axis Angle, Forefoot Var/Val, Forefoot Abd/Add, Hindfoot Var/Val, Midfoot Cavus/Planus, Troch Prominence Test Angle | |
| 6 | Gait Mechanics | Body Image and Self Esteem | Total | Lower Extremity Strength, Lower Extremity Static Motor Control, Lower Extremity Spasticity, Dynamic Motor Control, Diagnosis, Mean Pelvic Tilt, Range-of-Motion Pelvic Tilt, Min. Stance Hip Flexion, Mean Stance Hip Rotation, Init. Contact Knee Flexion, Min. Stance Knee Flexion, Range-of-Motion Knee Flexion, Max. Swing Knee Flexion, Mean Stance Knee Rotation, Init. Contact Ankle Dorsiflexion, Mean Stance Ankle Dorsiflexion, Mean Swing Ankle Dorsiflexion, Mean Stance Foot Progression, Normalized Step Length, Normalized Cadence, Age, Max Hip Flex, Max Hip Exten, Max Hip Abd (Knee Extended), Popliteal Angle (Unilateral, Max Knee Flex, Max Knee Exten, Extensor Lag, Max Ankle Dorsiflex (Knee Extended), Max Ankle Plantarflex, Troch Prominence Test Angle, Bimalleolar Axis Angle, Forefoot Var/Val, Forefoot Abd/Add, Hindfoot Var/Val, Midfoot Cavus/Planus | |
