# Supplementary material for: A model for understanding the causes and consequences of walking impairments
Source: PLoS One. 2022 Dec 28;17(12):e0270731. doi: 10.1371/journal.pone.0270731 (PMC9797092; doi:10.1371/journal.pone.0270731)
Supplement: S3 Appendix — (DOCX) [file pone.0270731.s004.docx]

# Appendix 3

| Diagnoses in “Other” category | N (%) |
| --- | --- |
| No Primary Diagnosis Listed | 22 (1.3%) |
| Adult CVA | 1 (<0.1%) |
| Amputee | 2 (0.1%) |
| Ankle disorder | 8 (0.5%) |
| Attention deficit hyperactivity disorder | 4 (0.2%) |
| Autism | 35 (2.0%) |
| Brain malformation | 52 (3.0%) |
| Brain tumor | 18 (1.0%) |
| Cancer | 9 (0.5%) |
| Central Hypotonia | 7 (0.4%) |
| Cerebral palsy | 0 (0%) |
| Childhood CVA | 87 (5.1%) |
| CNS infection/injury | 45 (2.6%) |
| Developmental delay | 61 (3.5%) |
| Developmental variants | 0 (0%) |
| Foot disorder | 139 (8.1%) |
| Fracture/trauma | 15 (0.9%) |
| Genetic disorder | 267 (16%) |
| Gross cognitive delay (MR) | 1 (<0.1%) |
| Hip disorder | 166 (9.7%) |
| Hydrocephalus | 32 (1.9%) |
| Knee disorder | 125 (7.3%) |
| Leg length inequality | 9 (0.5%) |
| Miscellaneous condition | 54 (3.1%) |
| Muscle/motor unit disorder | 21 (1.2%) |
| Myelomeningocele | 0 (0%) |
| Neurological unspecified | 102 (5.9%) |
| Neuromuscular unspecified | 136 (7.9%) |
| None | 11 (0.6%) |
| Normal | 20 (1.2%) |
| Other | 54 (3.1%) |
| Peripheral nerve damage | 8 (0.5%) |
| Questionable Diagnosis | 42 (2.4%) |
| Seizure disorder | 19 (1.1%) |
| Spinal cord injury | 41 (2.4%) |
| Spinal cord tumor | 15 (0.9%) |
| Spine disorder | 22 (1.3%) |
| Traumatic/acquired brain deficit | 0 (0%) |
| Unknown | 67 (3.9%) |
| Upper extremity injury | 2 (0.1%) |
